# Supplementary material for: Quantitative Theory for Critical Conditions of Like-Charge Attraction between Polarizable Spheres
Source: J Chem Theory Comput. 2025 Mar 17;21(6):2822–8. doi: 10.1021/acs.jctc.5c00144 (PMC11948334; doi:10.1021/acs.jctc.5c00144)
Supplement: Supplementary file 1 — ct5c00144_si_001.pdf [file ct5c00144_si_001.pdf]

# Supporting Information: Quantitative Theory for Critical Conditions of Like-Charge Attraction Between Polarizable Spheres

Yanyu Duan[†] and Zecheng Gan[*,†,‡]

[†] *Thrust of Advanced Materials, and Guangzhou Municipal Key Laboratory of Materials Informatics, The Hong Kong University of Science and Technology (Guangzhou), Guangzhou 511453, China.*

[‡] *Department of Mathematics, The Hong Kong University of Science and Technology, Hong Kong SAR, China*

E-mail: zechenggan@hkust-gz.edu.cn;zechenggan@ust.hk

## 1 Three-point image formula validation

In the Supporting Information (SI), we further validate our image formula by cross-comparing with the numerical results by Xu, et al, Phys. Rev. E 2013 (Ref[17] in the manuscript). Note that Ref[17] used the Gaussian units, so we correspondingly changed the units of our results to make it consistent with Ref[17]. Two sets of parameters are considered:

(i). $Q_1 = 1$, $Q_2 = 3$, relative permittivity $\epsilon_{\text{out}} = 1$, $\epsilon_1 = \epsilon_2 = \epsilon_{\text{in}}$, $a_1 = a_2 = 1$, $d = R - a_1 - a_2$, as shown in Fig. S1.

(ii). $Q_1 = Q_2 = 1$, relative permittivity $\epsilon_{\text{out}} = 1$, $\epsilon_1 = \epsilon_2 = \epsilon_{\text{in}}$, $a_1 = 1, a_2 = 3$, $d = R - a_1 - a_2$, as shown in Fig. S2.

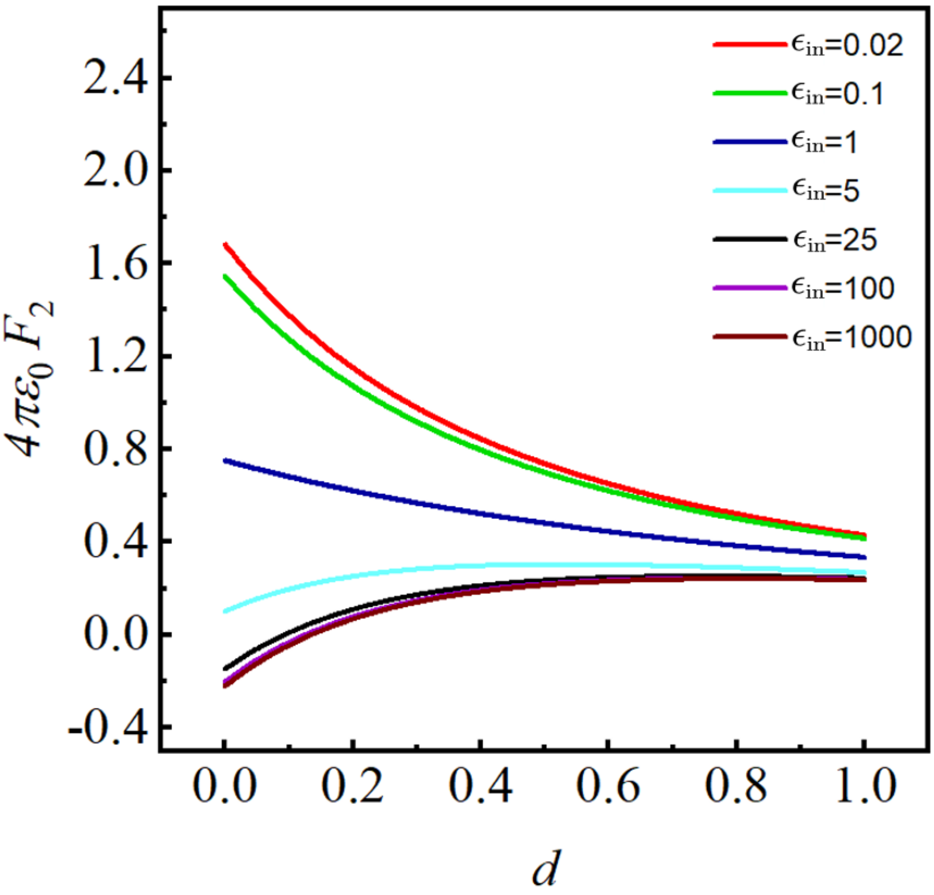


Figure S1: Forces between polarizable spheres under parameter set (i) using our three-point image formula. Benchmark results can be found from Fig.5 (left) of Ref[17].

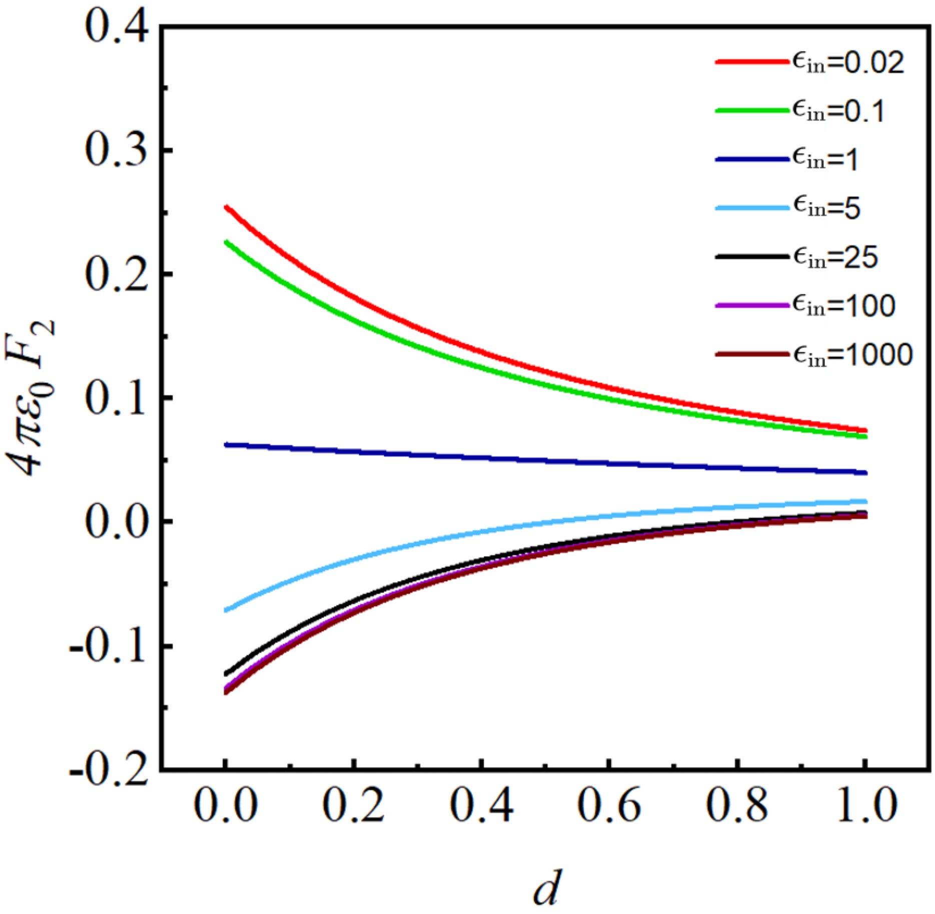


Figure S2: Forces between polarizable spheres under parameter set (ii) using our three-point image formula. Benchmark results can be found from Fig.5 (right) of Ref[17].

Note that there are some discrepancies between our results and Ref[17], mainly due to the model difference. In Ref[17], discrete surface charge distributions are considered, instead of central charges in our model. As a result, computed forces under our model are generally weaker at short separations, while the overall force behaviors are similar.

## 2 Critical condition validation

Here, we provide detailed data as a validation for the obtained critical conditions. We cross-compare our theoretical prediction of $R_c$ (or $t$ in dimensionless form) with numerical results obtained using a highly accurate hybrid method developed by one of the authors. All data comparing our theoretical prediction and benchmark numerical values are summarized below, inside Tables S1 (equal-sized case); and Table S2 (unequal-sized case). It is observed that, over all the system parameter settings considered here, our theory will always lead to a relative error of less than 1% in predicting the critical distance $R_c$, justifying the validity of our theory.

Table S1: A pair of equal-sized spheres with the same radii $a$, different charges $(Q_1, Q_2)$ and dielectric constants $(\epsilon_1, \epsilon_2)$, immersed in vacuum with permittivity $\epsilon_{\text{out}} = 1$. Recall that $t$ is defined as $a/R_c$, which is the dimensionless form of the critical separation distance predicted by our theory, and $t'$ is the numerical result obtained by a highly accurate hybrid method.

| $k_1\frac{Q_2}{Q_1} + k_2\frac{Q_1}{Q_2}$ | $Q_1$ | $Q_2$ | $\epsilon_1$ | $\epsilon_2$ | $a$ | $t$ | $t'$ | Err$\left(\sqrt{\frac{\sum_1^3 (t-t_i')^2}{3}}\right)$ |
|---|---|---|---|---|---|---|---|---|
| 8.682 | 9.3 | 1 | 24.916 | 24.916 | 1 | 0.36 | $\frac{1}{2.746}$ | 0.652% |
| | 21 | 2 | 10.073 | 10.073 | 1 | | $\frac{1}{2.713}$ | |
| | 9.852 | 1 | 4 | 15 | 1 | | $\frac{1}{2.732}$ | |
| 7.190 | 9 | 1 | 8.483 | 8.483 | 1 | 0.38 | $\frac{1}{2.563}$ | 0.667% |
| | 2 | 15 | 33.407 | 33.407 | 1 | | $\frac{1}{2.607}$ | |
| | 7.695 | 1 | 5 | 25 | 1 | | $\frac{1}{2.603}$ | |
| 5.481 | 6 | 1 | 16.997 | 16.997 | 1 | 0.41 | $\frac{1}{2.405}$ | 0.767% |
| | 3 | 21 | 7.598 | 7.598 | 1 | | $\frac{1}{2.376}$ | |
| | 5.858 | 1 | 8 | 22 | 1 | | $\frac{1}{2.411}$ | |
| 4.226 | 4.9 | 1 | 10.625 | 10.625 | 1 | 0.44 | $\frac{1}{2.236}$ | 0.502% |
| | 3 | 13 | 25.998 | 25.998 | 1 | | $\frac{1}{2.257}$ | |
| | 1 | 4.426 | 22 | 10 | 1 | | $\frac{1}{2.254}$ | |
| 3.286 | 4 | 1 | 7.819 | 7.819 | 1 | 0.47 | $\frac{1}{2.105}$ | 0.622% |
| | 3 | 14 | 5.121 | 5.121 | 1 | | $\frac{1}{2.086}$ | |
| | 3.280 | 1 | 6 | 30 | 1 | | $\frac{1}{2.130}$ | |

Table S2: Two spheres with the same charges ($Q_1 = Q_2 = Q$) and dielectric constants ($\epsilon_1 = \epsilon_2 = \epsilon_{\text{in}}$) while different size with radius $a_1$ and $a_2$, suspended in vacuum with permittivity $\epsilon_{\text{out}} = 1$. $t_1$, $t_2$ and $t_1^{'}$, $t_2^{'}$ are the theoretical values and numerical results, respectively.

| $\epsilon_{\text{in}}$ | $k$ | $Q$ | $a_1$ | $a_2$ | $t_1$ | $t_2$ | $t_1^{'}$ | $t_2^{'}$ | Err$\left(\sqrt{\frac{\left(t_1-t_1^{'}\right)^2+\left(t_2-t_2^{'}\right)^2}{2}}\right)$ |
|---|---|---|---|---|---|---|---|---|---|
| 10.776 | 0.830 | 5 | 1 | 0.25 | 0.64 | 0.16 | $\frac{1}{1.546}$ | $\frac{0.25}{1.546}$ | 0.499% |
| 3.016 | 0.502 | 16 | 1 | 0.1 | 0.7 | 0.07 | $\frac{1}{1.401}$ | $\frac{0.1}{1.401}$ | 0.979% |
| 1.457 | 0.186 | 20 | 1 | 0.125 | 0.8 | 0.1 | $\frac{1}{1.232}$ | $\frac{0.125}{1.232}$ | 0.589% |
